# Synthesis of di-rhamnolipids by the avirulent, mono-rhamnolipid producing strain *Pseudomonas aeruginosa* ATCC 9027

**DOI:** 10.1007/s10529-024-03527-7

**Published:** 2024-09-03

**Authors:** Abigail González-Valdez, Paola G. Vázquez-Bueno, Jessica Hernández-Pineda, Gloria Soberón-Chávez

**Affiliations:** 1 Departamento de Biología Molecular y Biotecnología, Instituto de Investigaciones Biomédicas, Universidad Nacional Autónoma de México, Apdo. Postal 70228, C. P. 04510 University City, CDMX Mexico; 2 Departamento de Infectología E Inmunología, Instituto Nacional de Perinatología, SSA, C. P. 11000 Mexico City, CDMX Mexico

**Keywords:** Avirulent *Pseudomonas aeruginosa*, Biosurfactants, di-rhamnolipids production, Quorum sensing

## Abstract

To construct a derivative of the avirulent *Pseudomonas aeruginosa* ATCC 9027 that produces high levels of di-rhamnolipid, that has better physico-chemical characteristics for biotechnological applications than mono-rhamnolipid, which is the sole type produced by ATCC 9027. We used plasmids expressing the *rhlC* gene, which encodes for rhamnosyl transferase II that transforms mono- to di-rhamnolipids under different promoters and in combination with the gene coding for the RhlR quorum sensing regulator, or the mono-rhamnolipid biosynthetic *rhlAB* operon. The plasmids tested carrying the *rhlC* gene under the *lac* promoter were plasmid p*rhlC* and p*rhlRC*, while p*rhlAB-R–C* expressed this gene from the *rhlA* promoter, forming part of the artificially constructed *rhlAB-R–C* operon. We measured rhamnolipds concentrations using the orcinol method and determined the proportion of mono-rhamnolipids and di-rhamnolipids by UPLC/MS/MS. We found that the expression of *rhlC* in *P. aeruginosa* ATCC 9027 caused the production of di-rhamnolipids and that the derivative carrying plasmid p*rhlAB-R–C* gives the best results considering total rhamnolipids and a higher proportion of di-rhamnolipids. A *P. aeruginosa* ATCC 9027 derivative with increased di-rhamnolipids production was developed by expressing plasmid p*rhlAB-R–C*, that produces similar rhamnolipids levels as PAO1 type-strain and presented a higher proportion of di-rhamnolipids than this type-strain.

**Supplementary Information:**

The online version contains supplementary material available at 10.1007/s10529-024-03527-7.

## Introduction

*Pseudomonas aeruginosa* is an environmental bacterium and an opportunistic pathogen that represents an important health hazard due to its production of virulence factors and high intrinsic and acquired antibiotic resistance (Jurado-Martín et al. 2021).

Biosurfactants (BS) are amphiphilic molecules produced by microorganism that have a wide range of industrial potential applications, but in contrast with chemically synthetized surfactants they are biodegradable and nontoxic (Abbot et al. 2022). *P. aeruginosa* is the best natural producer of the BS rhamnolipids (RL) (Toribio et al. 2010), which is one of the two commercially available BS (Soberón-Chávez et al. 2021); the other being sophorolipids produce by the yeast *Starmerella bombicola* (Pal et al. 2023).

*P. aeruginosa* produces two types of RL, mono-RL which consists of a fatty acid dimer (3-(3-hydroxyalkanoyloxy) alkanoic acids or HAAs) -mainly of β-hydroxy decanoate molecules (C10); and di-RL, which includes an additional rhamnose moiety. The RhlA enzyme catalyzes the formation of HAAs using a CoA-link fatty acid precursor (Abdel-Mawgoud et al. 2014), while RhlB uses HAAs and dTDP-L-rhamnose as substrates to produce mono-RL. In turn, RhlC transfers a rhamnose from dTDP-L-rhamnose to mono-RL to produce di-RL (Rahim et al. 2001). Mono-RL and di-RL BS have different physico-chemical properties (Esposito et al. 2023), so the proportion of their production affects their biotechnological applications (Wu et al. 2024).

*P. aeruginosa* produces RL in a coordinate manner with difference virulence associated traits by a complex regulatory network that is called quorum sensing (QS) and consists of three systems (Las, Rhl and Pqs systems) arranged in a hierarchical manner (Williams and Cámara 2009). LasR and RhlR are the transcriptional regulators of the Las and Rhl systems, and both belong to the LuxR family that interact with acyl-homoserine lactone autoinducers (AI), whereas PqsR, which belongs to the LysR family, is the activator of the Pqs system and interacts with alkyl-4(1H)-quinolones (AQs) AI. LasR forms a complex with 3-oxo-dodecanoyl-homoserine lactone (3O-C12-HSL) produced by the LasI enzyme and activates the transcription of several genes encoding virulence factors, such as *lasB*, that codes for elastase, and also of *rhlR, lasI*, *rhlI,*and *pqsR.* In turn, *rhlR* encodes the second QS transcriptional regulator, RhlR, while the product of *rhlI* produces butanoyl-homoserine lactone (C4-HSL), which is the AI that interacts with RhlR to activate the *rhlAB-R* operon (Croda-García et al. 2011) and *rhlC* which is the second gene of an operon with PA1131 (Rahim et al. 2001) that is not involved in RL synthesis or transport (Wittgens et al. 2017) and the *phz* genes involved in the production of the toxin pyocyanin (Mavrodi et al. 2001). PqsR modulates QS by activating the transcription of the *pqsABCDE* operon that encodes the enzymes responsible for the synthesis of AQs. This regulon acts mainly through the modulation of RhlR activity by PqsE affecting the production of the toxin pyocyanin (Groleau et al. 2020; Borgert et al. 2022).

The main draw-back for RL production using *P. aeruginosa* at an industrial scale is the pathogenicity of this bacterium (Jurado-Martín et al. 2021). To circumvent this problem different microorganisms have been used as heterologous host for the expression of *P. aeruginosa* biosynthetic genes (Wittgens and Rosenau 2020). In particular, the model for heterologous RL production by *Pseudomonas putida* KT2440 has been very well developed (Filbig et al. 2023; Noll et al. 2024). Recently, we described the heterologous production of RL in the innocuous soil bacterium *Pseudomonas chlororaphis* ATCC 9446 that besides expressing *P. aeruginosa* RL biosynthetic genes, expressed the *P. aeruginosa* QS transcriptional regulator RhlR which is activated by the *P. chlororaphis* naturally produced AI (González-Valdez et al. 2024a). Thus the *P. chlororaphis* system for heterologous production of RL has the advantage of being positively autoregulated and do not need the addition of a chemical inducer. However, much work is still needed to optimize the production of RL by genetically manipulating *P. chlororaphis.*

Metabolic engineering strategies to construct *P. aeruginosa* derivatives that hyper produced RL have been reported (Gutiérrez-Gómez et al. 2018) using a non-virulent derivative of the PA14 type strain (Gutiérrez-Gómez and Soberón-Chávez 2024). In addition, we described the production of high mono-RL levels in the non-virulent *P. aeruginosa* ATCC 9027 strain by the expression of a constructed plasmid encoding the biosynthetic *rhlAB* operon containing *rhlR* (forming the *rhlAB-R* operon) (Grosso-Becerra et al. 2016).

The ATCC 9027 strain forms part of the highly divergent *P. aeruginosa* phylogroup 3 (Quiroz-Morales et al. 2023), which has even been claimed to be a new species called *Pseudomonas pararuginosa* (Rudra et al. 2022). This strain is completely avirulent (Grosso-Becerra et al. 2016) and has been shown to have different mutations affecting the regulation of virulence factors expression (Quiroz-Morales et al. 2023) but can produce RL (Grosso-Becerra et al. 2016). Thus, as the ATCC 9027 avirulent phenotype depends on several gene mutations (García-Reyes et al. 2021), this strain is a very good model for RL production and other potential biotechnological applications, since the possibility of its reversion to a virulent phenotype is not plausible.

The heterologous production of di-RL has been reported using different bacteria as hosts to express *rhlA, rhlB* and *rhlC P. aeruginosa* genes. In the case of *Escherichia coli, rhlC* was expressed under different promoters in the presence of *rhlAB* operon expressed from an inducible promoter and small amounts of both mono- and di-RL were produced (Du et al. 2017); while we recently reported that the expression in *Pseudomonas chlororaphis* ATCC 9446 of the artificially constructed *rhlAB-R–C* operon results in the production of mainly di-RL (González-Valdez et al. 2024a). However, the highest di-RL yields have been obtained using *P. putida* T2440 expressing plasmid pSynPro8oT_rhlAB (Noll et al. 2024).

All strains belonging to *P. aeruginosa* phylogroup 3 have a deletion of the *rhlC* gene (Quiroz-Morales et al. 2022), and hence they only produce mono-RL. In this context, the aim of this work was to determine whether di-RL production could be achieved in the ATCC 9027 background by the expression of *rhlC*, and whether the expression of this gene in the presence and absence of the *rhlR* gene, and or the *rhlAB* operon causes an increase in the production of these BS, specially of di-RL. We found that the solely expression of *rhlC* from the *lac* promoter that in *Pseudomonas* is constitutive, results in di-RL production. The highest levels of di-RL were obtained when the artificial operon *rhlAB-R–C* was expressed, yielding a much higher proportion of di-RL than the PAO1 strain. These results show that the avirulent ATCC 9027 *P. aeruginosa* strain has potential biotechnological applications for mono-RL and di-RL production.

## Methods

### Microbiological procedures

Strains and plasmids, and the oligonucleotides used in this work are shown in Supplementary information (Tables S1 and S2, respectively).

*P. aeruginosa* PAO1, used as positive control in this work, and ATCC 9027 derivatives studied in this work were routinely cultured in PPGAS medium (Zhang and Miller 1992) to measure RL and pyocyanin, for 24 h at 37 °C. Overnight cultures were grown in LB medium (Miller 1972) at 37 °C.

### Construction of the plasmids encoding *rhlC* alone or in combination with *rhlR* or *rhlAB-R*.

Standard molecular biology techniques were used in this work (Sambrook et al. 1989). The *rhlC* gene with its ribosome binding site was amplified from PAO1 genomic DNA as a template using primers FwH3rhlC and rhlCReH3 that contain a *Hind*III restriction site (Table S2). The plasmids encoding *rhlC* that were used to evaluate the production of di-RL (Table S1) were constructed by introducing this PCR product digested with the same restriction enzyme into the following plasmids: pUCP24 (West et al. 1994) resulting in p*rhlC* (Table S1) and pJMG1-*rhlR* (Grosso-Becerra et al. 2016). Plasmid p*rhlAB-R–C* (González-Valdez et al. 2024a) was constructed in the same way using pJMG4-*rhlAB-R* (Grosso-Becerra et al. 2016) as starting material. In all these cases the plasmids that contain *rhlC* in the correct direction when cloned in the *Hind*III restriction site were detected by the production of di-RL in strain ATCC 9027 and they were corroborated by sequencing.

### Measurement of RL concentration using the orcinol method

The orcinol method was used to quantified RL as rhamnose equivalents, as described previously (González-Valdez et al. 2024b). Briefly, 333 μL of the filtered supernatant was extracted twice with 1 mL of diethyl ether. The solvent was evaporated to dryness and dissolved in 1 mL of deionized water. Then, 900 μL of a solution containing 0.19% orcinol (in 53% sulfuric acid) was added to 100 μL of each sample. These solutions were heated at 80 °C in a water bath for 30 min and cooled for 15 min at room temperature, and the absorbance at 421 nm was measured. The concentration of RL was determined by comparing the data with L-rhamnose standards between 0 and 50 μg/mL. The orcinol method to measure RL has been recently shown to be reproducible and accurate, compared with the detection of these BS by UPLC/MS/MS (González-Valdez et al. 2024b).

### Determination of pyocyanin

The blue-green toxin pyocyanin was determined by measuring the culture supernatant absorbance at 695 nm (Groleau et al. 2020). The measured absorbance was divided by the optical density at 600 nm of the culture.

### UPLC-MS/MS conditions for analysis of RL proportion.

Di-RL and m-RL were analyzed employing an ultra-high performance liquid chromatography coupled with tandem mass spectrometry (UPLC-MS/MS) method standardizing in house. An ACQUITY UPLC H-class system coupled to a Xevo TQ-S tandem mass detector equipped with an ESI source from Waters (Waters Corp., Milford, MA, USA) was employed. The chromatographic separation was made of 35 °C on an Acquity BEH C18 column (2.1mmx50mm, 1.7 µm) under gradient conditions using water (%A) and acetonitrile (%B), both with formic acid (0.1%) as mobile phase. The initial conditions were 90% A:10% B for 1.0 min, then a linear gradient to 10% A:90% B by 6.0 min followed by change to initial conditions until 8.0 min, and then completed a total run time of 10.0 min. The volume injection was 4-µL. The samples were prepared in methanol grade LC–MS. Spectrometric mass detection was obtained employing electrospray negative ion (ESI-) in a selected ion recording (SIR) mode. Desprotonated ions (m/z) were set according to the reported data by Rudden et al. (2015) as following, for Di-RLs: Di-C_8_-C_10_/DI-C_10_-C_8_ (m/z 621); Di-C_10_-C_10_ (m/z 649); Di-C_10_-C_12:1_/DI-C_12:1_-C_10_ (m/z 675) and Di-C_10_-C_12_/DI-C_12-_C_10_ (m/z 677). For Mono-RLs: Mono-C_8_-C_10_/DI-C_10_-C_8_ (m/z 475); Mono-C_10_-C_10_ (m/z 503); Mono-C_10_-C_12:1_/DI-C_12:1_-C_10_ (m/z 529) and Mono-C_10_-C_12_/DI-C_12-_C_10_ (m/z 531). Data acquisition and peak integration were performed with MassLynx version 4.1 software (Waters Corp.)

Using the proportion thus determined, the approximate μM concentration of each type of RL was calculated considering that mono-RL have 1 rhamnose per molecule and di-RL have two rhamnoses per molecule. Strain PAO1 was considered to produce 20% of mono-RL and 80% of di-RL, strain ATCC 9027/pr*hlC* 10% of mono-RL and 90% of di-RL, while strains ATCC 9027/pr*hlRC* and ATCC 9027/pr*hlAB-R–C* were consider producing 5% of mono-RL and 95% of di-RL.

## Results and discussion

### The expression of *rhlC* in the ATCC 9027 background results in di-RL production

**Fig. 1:**
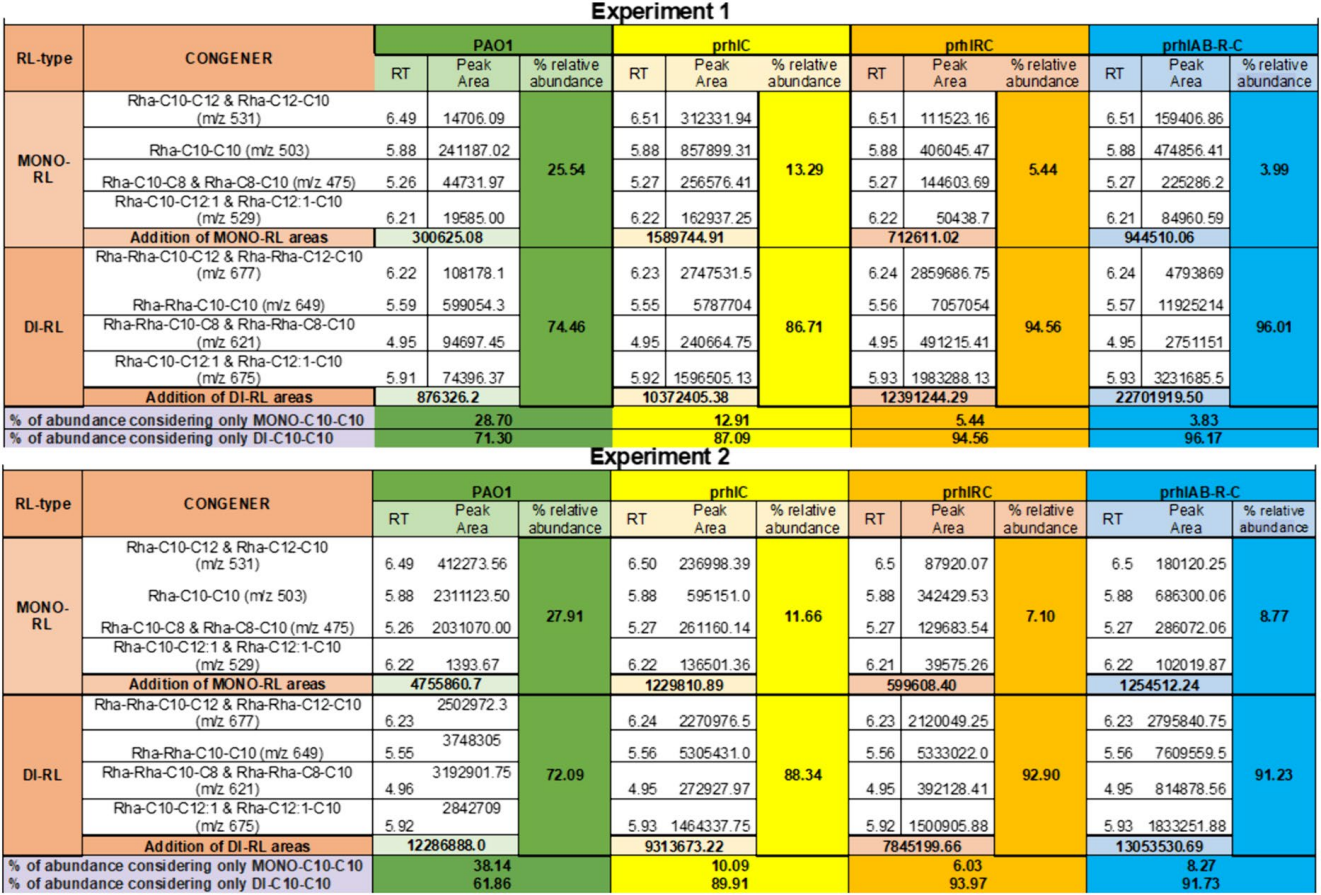
Proportion of mono-RL and di-RL congeners in two independent cultures of different strains expressing the rhlC gene. The prportion of each type of congener was measured using UPLC/MS/MS as decribed in the materials and methods section. Two independent experiments with two technical replicas are shown. RT means retention time of chromatographic peak; m/z is the mass to charge ratio

The expression of plasmid p*rhlC* in strain ATCC 9027 causes the production of di-RL, while the total amount of RL is slightly decreased (Table 1). The determination by UPLC/MS/MS of the proportion of mono- and di-RL produced by strain ATCC 9027/p*rhlC* shows that 87.5% corresponds to di-RL, while 12.5% of RL remains as mono-RL (Fig. 1). These results suggest that the constitutively expressed RhlC enzyme catalyzes the conversion from mono-to di-RL of a little less than 90% of the mono-RL molecules naturally produced by ATCC 9027 and causes a slight reduction of the rate of synthesis of mono-RL, since strain ATCC 9027/pUCP-24 produced 303 mM of mono-RL and strain ATCC 9027/p*rhlC* only 250 mM of the sum of mono-RL and di-RL. The observed reduction might be due to the limitation of dTDP-L-rhamnose for both mono-RL and di-RL synthesis.

**Table 1:** Estimation of the effect of different plasmids encoding *rhlC* on the production of RL in ATCC 9027 strain

Strain	Rhamnose in RL^+^ (μg/ml)	Rhamnose in RL (mM)	Proportion of mono-RL; di-RL* (%)	Approximate mono-RL; di-RL (mM)	Approximate RL produced (mM)
PAO1	120.4 ± 12.5	745.67 ± 76.4	26.7; 73.3	83; 332	415
ATCC 9027	47.36 ± 5.94	288.5 ± 36.18	100; 0	288; 0	288
ATCC 9027/ pUCP24	49.8 ± 8.08	303.36 ± 49.22	100; 0	303; 0	303
ATCC 9027/ p*rhlC*	77.9 ± 13.08	474.54 ± 79.68	12.5; 87.5	25; 225	250
ATCC 9027/ pJMG1**-***rhlR*	90.4 ± 11.72	550.68 ± 71.39	100; 0	550; 0	550
ATCC 9027/ p*rhlRC*	95.24 ± 6.3	580.11 ± 38.38	6.3; 93.7	15; 285	300
ATCC 9027/ pJMG4-*rhlAB-R*	107.36 ± 4.8	654 ± 29.24	100; 0	654; 0	654
ATCC 9027/ p*rhlAB-R–C*	123.01 ± 9.54	749.32 ± 58.11	6.4; 93.6	20; 400	420

The concentration of rhamnose equivalents in RL was determined using the orcinol method

^*^The proportion was determined by UPLC/MS/MS and we use the mean of the values of the experiments shown in Fig. 1

### The combined expression of *rhlR* and *rhlC* has no effect on total RL production.

It has been shown that the expression of the RhlR transcriptional regulator in ATCC 9027 caused a considerable increase in mono-RL production (Grosso-Becerra et al. 2016) (Table 1), presumably due to the increased expression of the chromosomally encoded *rhlAB* operon, which is directly activated by RhlR (Croda-García et al. 2011), and an increased dTDP-L-rhamnose production caused by the induction of the *rmlBDAC* operon encoding the enzymes involved in the synthesis of this activated sugar, which is also induced by RhlR (Aguirre-Ramírez et al. 2012).

**Fig. 2:**
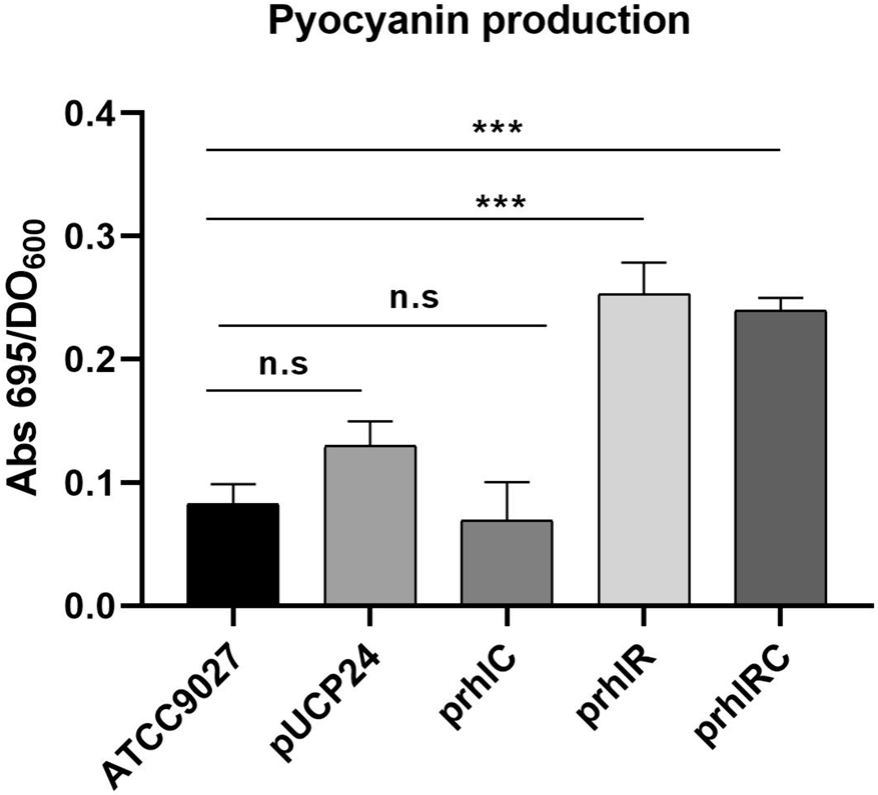
Production of pyocyanin by strain ATCC 9027 carrying different plasmids. The production of the blue-green pigment pyocyanin was determined at 695 nm and it was standardized for the growth of the culture determined at 600 nm. Bars correspond to strain ATCC 9027 without plasmids (ATCC9027) or carrying one of the following plasmids: pUCP24; prhlC; prhlR; or prhlRC. Statistical analysis was determined by Student’s t test (***,p ≤ 0.001, ns: not significant p > 0.05)

However, expressing *rhlC* together with *rhlR* from a plasmid in strain ATCC 9027 (ATCC 9027/p*rhlRC*) does not result in an increase of total RL production compared to the strain carrying the empty vector pUCP24, even though most of the RL produced are in the form of di-RL (Table 1). These results could be explained if *rhlR* was not expressed when forming part of the plasmid p*rhlRC,* even though *rhlC* is effectively expressed from this plasmid since di-RL are produced (Table 1). However, this is not the case since the production of pyocyanin, the *P. aeruginosa* virulence factor that is encoded by the *phz* genes that are activated by RhlR transcriptional factor (Mavrodi et al. 2001), is increased in the presence of plasmid p*rhlRC* to the same levels as when plasmid p*rhlR* is expressed in strain ATCC 9027, and not affected by plasmid p*rhlC* (Fig. 2). Thus, the most likely explanation for the lack of an apparent effect of plasmid p*rhlRC* in total RL production is that when both *rhlR* and *rhlC* are constitutively expressed under the *lac* promoter, a substrate for the biosynthetic RL enzymes RhlA, RhlB or RhlC becomes limiting. The possibly limiting metabolites are the CoA-linked fatty acid substrate of RhlA, HAAs, or dTDP-L-rhamnose.

The UPLC/MS/MS analysis of the RL produced by ATCC 9027/p*rhlRC* shows that 93.7% are di-RL (Fig. 1), thus it is probable that in this condition, the HAAs produced by RhlA are limiting for production of similar RL levels as strain ATCC 9027/pJMG1-*rhlR*, since the limiting step seems to be the production of mono-RL and the production of RhlA fatty acid substrate does not seem to be activated by RhlR as is the production of dTDP-L-rhamnose (Aguirre-Ramírez et al. 2012).

### The expression of *rhlC* in strain ATCC 9027 as part of the artificial operon *rhlAB-R–C* causes a similar total RL production as PAO1 strain, but with a much higher di-RL proportion

The expression in ATCC 9027 of the *rhlAB-R* operon from a plasmid was reported to cause the highest increment in mono-RL production (Grosso-Becerra et al. 2016) (Table 1) and considering that RL production of ATCC 9027/p*rhlRC* might be limited by RhlA activity, we explore whether ATCC 9027/p*rhlAB-R–C* could produce higher di-RL levels.

Our results show that indeed di-RL levels were increased in ATCC 9027/p*rhlAB-R–C* compared to ATCC 9027/p*rhlRC* (Table 1), but the total RL levels obtained in this strain were significantly lower than those produced by ATCC 9027/p JMG4-*rhlAB-R* (Table 1). These results suggest that di-RL production is limited in strain ATCC 9027/p*rhlAB-R–C* by dTDP-L-rhamnose or the Co-A linked precursor of HAAs availability, or by the activity of *rhlC* that might not be expressed to the same levels as other RL biosynthetic enzymes, since the only copy is the one encoded in plasmid p*rhlAB-R–C*. These possibilities remain to be determined and from the results obtained, new strategies to construct an ATCC 9027 derivative producing higher di-RL levels could be developed.

Strain ATCC 9027/p*rhlAB-R–C* produces a similar concentration of total RL as PAO1 strain (Table 1), but most of the RL produced is di-RL (93.6%) which has better physico-chemical characteristics for some biotechnological applications and has the important advantage of being completely avirulent.

In addition, an important biotechnological advantage of the di-RL producing ATCC 9027 derivatives reported in this work is that the production of this BS is not dependent on the addition of a chemical inducer as is required for mono-RL and di-RL production using the *P. putida* KT2440 model (Noll et al. 2024), since we used either constitutive lac promoter or the *rhlA* promoter that is positively regulated by RhlR (chromosomal and plasmid encoded) and the endogenously produced C4-HSL.

In conclusion, the use of strain ATCC 9027 expressing the p*rhlAB-R–C* plasmid described in this work, is a good alternative to produce di-RL using a completely avirulent strain that does not require the addition of chemical inducers, since it makes use of the indigenous *P. aeruginosa* QS regulatory network.

## Supplementary Information

Below is the link to the electronic supplementary material.

Supplementary file1 (PDF 169 KB)
