# Supplementary material for: Synthesis of di-rhamnolipids by the avirulent, mono-rhamnolipid producing strain Pseudomonas aeruginosa ATCC 9027
Source: Biotechnol Lett. 2024 Sep 3;46(6):1163–70. doi: 10.1007/s10529-024-03527-7 (PMC11550238; doi:10.1007/s10529-024-03527-7)
Supplement: Supplementary file 1 — Supplementary file1 (PDF 169 KB) [file 10529_2024_3527_MOESM1_ESM.pdf]

SUPPLEMENTARY INFORMATION

TITLE: Production of di-rhamnolipids by the avirulent, mono-rhamnolipid producing *Pseudomonas aeruginosa* ATCC 9027 strain.

AUTHORS: Abigail González-Valdez[1], Paola G. Vázquez-Bueno[1], Jessica Hernández-Pineda[2], Gloria Soberón-Chávez*[1].

AFFILIATIONS: [1]Departamento de Biología Molecular y Biotecnología, Instituto de Investigaciones Biomédicas, Universidad Nacional Autónoma de México, Ciudad Universitaria, Apdo. Postal 70228, C. P. 04510, CDMX, México.

[2]Departamento de Infectología e Inmunología. Instituto Nacional de Perinatología, SSA, C. P. 11000, CDMX, México

*Corresponding author, email address gloria@biomedicas.unam.mx. ORCID 0000-0002-0282-3882.

Keywords: Di-rhamnolipids production, avirulent *Pseudomonas aeruginosa*, Biosurfactants, quorum sensing.

Type of paper: Microbial and Enzyme Technology

Table S1. List of strains and plasmids used in this work.

**Strains**		
**Name**	**Relevant genotype/phenotype**	**Reference**
Escherichia coli		
DH5α	*lacZ*ΔM15 Δ(*lacZYA*-*argF*)U169 *recA1 endA1 hsdR17* [rKmK+] *supE44 thi-1 gyrA relA1*	Sambrook et al., (1989)
Pseudomonas aeruginosa		
PAO1	Wild type strain used in this work.	Holloway, 1955
ATCC 9027	Wild type strain used in this work.	ATCC Collection
**Plasmids**		
**Name**	**Relevant genotype/phenotype**	**Reference**
pUCP24	Cloning vector (Gm^R)	West SE. *et al.* (1994)
pJMG4-*rhlAB-R*	pUCP24 derived plasmid with a 2.7 kb fragment containing PAO1 *rhlAB-R* operon	Grosso-Becerra *et al.* (2016)
pJMG1-*rhlR*	pUCP24 derived plasmid with a .7 kb fragment containing PAO1 *rhlR* (Gm^R)	Grosso-Becerra *et al*. (2016)
p*rhlAB-R-C*	Constructed by ligation the 1.0-kb *rhlC* gene cloned at *Hind*III site in pJGM4-rhlABR plasmid (Gm^R)	González-Valdez *et al.* (2024)
pr*hlC*	Constructed by ligation the 1.0-kb *rhlC* gene cloned at *Eco*R1-*Hind*III site in pUCP24 plasmid (Gm^R)	This work

p*rhlRC*	Constructed by ligation the 1.0-kb *rhlC* gene cloned at *Hind*III site in pJGM1-rhlR plasmid (Gm^R)	This work

Table S2. Oligonucleotides used in this work

**Name**	**Sequence (5´- 3´)**	**Refence**
rhlCReH3	GCGTTTAAGCTTCTAGGCCTTGGCCTTGCCGG	González-Valdez *et al.* (2024)
FwH3rhlC	CGGGCTAAGCTTGGCCTGGCAACTTCGACCTA	González-Valdez *et al.* (2024)
rhlCFwE1	AGAAGAGAATTCATGGACCGGATAGACATGGG	This work

The *Hind*III restriction site is underlined.

REFERENCES

González-Valdez A, Escalante A, Soberón-Chávez G. (2024) Heterologous production of rhamnolipids in Pseudomonas chlororaphis subsp chlororaphis ATCC 9446 based on the endogenous production of N-acyl-homoserine lactones. Microb Biotechnol. MBT 17(1): e14377.

Grosso-Becerra MV, González-Valdez A, Granados-Martínez M, Morales E, Servín-González L, et al. (2016). *Pseudomonas aeruginosa* ATCC9027 is a non-virulent strain suitable for mono-rhamnolipids production. Appl Microbiol Biotechnol, 100(23): 9995-10004.

Holloway BW. (1955) Genetic recombination in *Pseudomonas aeruginosa*. J Gen Microbiol 13:572–581.

West SE, Schweizer HP, Dall C, Sample AK, Runyen-Janecky LJ. (1994) Construction of improved Escherichia-Pseudomonas shuttle vectors derived from pUC18/19 and sequence of the region required for their replication in Pseudomonas aeruginosa. Gene. 48(1):81-86.

Sambrook J, Fritsch EF, Maniatis T. (1989) Molecular cloning: a laboratory manual, 2nd ed. Cold Spring Harbor Laboratory Press.
